# Dietary Carbohydrate Intake, Dietary Glycemic Load and Outcomes of In Vitro Fertilization: Findings from an Observational Italian Cohort Study

**DOI:** 10.3390/nu12061568

**Published:** 2020-05-28

**Authors:** Stefania Antonia Noli, Elena Ricci, Sonia Cipriani, Stefania Ferrari, Marta Castiglioni, Irene La Vecchia, Edgardo Somigliana, Fabio Parazzini

**Affiliations:** 1 Department of Clinical Sciences and Community Health, Università degli Studi di Milano, 20122 Milan, Italy; martacastiglioni145@gmail.com (M.C.); irene.lavecc@gmail.com (I.L.V.); dadosomigliana@yahoo.it (E.S.); 2 Fondazione IRCCS Ca’ Granda Ospedale Maggiore Policlinico, 20122 Milan, Italy; ed.ricci@libero.it (E.R.); son.cipriani@gmail.com (S.C.); stefania.ferrari@policlinico.mi.it (S.F.)

**Keywords:** diet, glycemic load, glycemic index, carbohydrate, in vitro fertilization, assisted reproductive techniques, fertility

## Abstract

In recent decades, increasing attention has been paid to the influence of diet on reproductive health. Carbohydrates in diet affect glucose metabolism and multiple evidences showed the key role of insulin sensitivity in regulating female fertility. We designed a prospective cohort study to investigate the relation between dietary carbohydrate intake, glycemic load (GL) and the outcomes of assisted reproduction. A population of 494 female partners of couples referring to an Italian Fertility Center and eligible for in vitro fertilization (IVF) were enrolled in the study. On the day of the oocyte retrieval, information on their diet was obtained using a validated food frequency questionnaire (FFQ). We calculated the relative risk and 95% confidence interval of embryo transfer, clinical pregnancy and live birth according to the following dietary exposures: GL, glycemic index (GI) as well as the daily carbohydrate and fiber intake. A multiple regression model was used to account for the confounders. After adjusting for age, college degree, body mass index (BMI), leisure physical activity and previous assisted reproduction techniques (ART) cycles, no significant association was observed between the considered dietary exposures and the IVF outcomes. The roles of GL, carbohydrate intake and GI were assessed in strata of the cause of infertility and body mass index and no relation emerged in this further analysis. We found no clear association between the dietary carbohydrate quantity and quality and IVF outcomes in a cohort of infertile Italian women.

## 1. Introduction

In recent decades, increasing attention has been paid to the influence of lifestyle on reproductive health, highlighting the impact of body weight, physical activity and dietary habits on fertility [1,2,3]. In this regard, the tight connection between energy metabolism and reproduction, especially in female animals, plays a key role and multiple evidences have linked glucose metabolism and insulin sensitivity to ovulatory function and fertility [4,5]. Recent studies suggested that insulin has a direct regulatory effect on ovarian physiology and it is involved in the response of ovarian follicular cells to gonadotropins [6,7,8]. Consequently, several authors have investigated ovulation and, in general, the reproductive function of women with polycystic ovary syndrome (PCOS) and of obese women [9,10,11], two subgroups of patients characterized by hyperinsulinemia and insulin resistance. In these conditions, higher insulin levels seemed to favor a disruption of the ovarian steroidogenesis, with the increased secretion of ovarian androgens and the impairment of follicular and oocyte development [7,8,12].

Referring to in vitro fertilization (IVF), these observations may help to explain, on the one hand, the higher percentages of low-quality oocytes and consequent lower fertilization rate reported in PCOS women [13], while on the other hand, the higher frequency of poor ovarian response to follicle-stimulating hormone (FSH)-stimulation and the decreased E2 production described in obese women [8,14].

Both the quality and quantity of dietary carbohydrates affect glucose metabolism and insulin sensitivity, not only in diabetics, obese and PCOS women, but also in healthy subjects [15,16,17]. Along this line, in the Nurses’ Health Study II (NHS II), the total carbohydrate intake and the dietary glycemic load (GL) were positively related to ovulatory infertility in otherwise healthy women [18]. Furthermore, recent evidences have shown that low carbohydrate and low GL diets can lead to a reduction in circulating insulin levels, to an improvement in hormonal imbalance and lastly to the resumption of ovulatory function with improved chances of both natural and assisted conception [19,20].

Despite the presence of growing reports about the key role of insulin sensitivity in regulating female fertility, there are few data available on the impact of carbohydrate consumption and of glycemic load on the success rate of assisted reproduction.

To provide further information on the association between dietary carbohydrate quantity and quality (i.e., glycemic load, glycemic index (GI)) and IVF outcomes, we examined the data from an Italian prospective cohort study including women of infertile couples referring to an Academic Fertility Center.

## 2. Materials and Methods

From September 2014 to December 2016, infertile couples referring to the Infertility Unit of Fondazione IRCCS Ca’ Granda Ospedale Maggiore Policlinico located in Milan (northern Italy), and eligible for in vitro fertilization, were invited to take part in an ongoing prospective cohort study investigating the influence of lifestyle and dietary habits on assisted reproduction techniques (ART) outcomes [21,22]. The Ethical Review Board of the Fondazione approved the study protocol. All the procedures were in accordance with the Helsinki Declaration and participants subscribed written informed consent before entering the study.

The time period between the proposal of the study and couples’ interview was less than 1 month. On the day of the oocyte retrieval, a specially trained staff interviewed both partners of the participating couples using a standard questionnaire to collect information on socio-demographic and anthropometric features, health and reproductive history and lifestyle habits. Couples who did not speak fluent Italian were excluded from the study, as the administered questionnaires were validated for Italian speaking subjects only. The present paper exclusively reported on evidences from the female partners in relation to the outcome of the IVF cycle that was ongoing at the time of the interview.

The overall participation rate was close to 95% (Figure S1). Such a high rate is mainly attributable to the non-sensitive content of the questions and to the fact that the couples were interviewed during the downtime spent in the hospital after the oocyte retrieval (commonly between 2 and 5 h).

Dietary habits were investigated using a reproducible and previously validated food frequency questionnaire (FFQ), which assessed the average weekly consumption of 78 food items or food groups [23,24,25]. This questionnaire had been previously used in several epidemiological studies in the gynecological and obstetric field [26,27,28]. As described in detail elsewhere [21,22], patients were asked to report about their usual weekly food consumption in the last year. An intake lower than once per week, but at least once per month, was coded 0.5 per week. The questionnaire also allowed estimating the total energy intake and the mineral, macro- and micronutrient intake using the most recent update of an Italian food consumption database [29]. We did not report about folic acid supplementation as it is routinely prescribed to all women seeking a pregnancy. We described in detail in previous publications how information on alcohol and caffeine intake and tobacco smoking was collected and categorized [21,22]. The reproducibility and validity of alcohol consumption have been previously assessed with satisfactory results [30,31]. Occupational physical activity (PA) was classified as heavy (or very heavy), light/moderate, primarily standing or primarily sitting, while leisure PA was evaluated in hours per week: <2, between 2 and 4, or ≥5. The type and intensity of leisure PA were not investigated.

### 2.1. Calculation of Glycemic Index and Glycemic Load

For each food containing carbohydrates, the glycemic index (GI) was expressed as the percentage of the glycemic response aroused considering white bread as standard food and referring to the International Glycemic Index tables [32]. The average daily glycemic index of every subject was calculated by summing the products of the glycemic index of one serving of each food times the average number of servings of that food consumed by the person per week, divided by the weekly available carbohydrates [32,33]. To take into proper account Italian cooking habits (i.e., pasta al dente), Italian publications were used as a reference for some recipes [34]. Food items for which a glycemic index was not available were assigned the value of the closest food comparable (i.e., tangerines were assigned the glycemic index of oranges). The daily average glycemic load was computed by summing the products of the glycemic index of one serving of each food times the average number of servings of that food consumed by the subject per week.

### 2.2. Clinical Management

Patients’ clinical management was described in detail elsewhere [35]. Before IVF, they underwent ovarian reserve testing and they were assigned one of the ovarian stimulation protocols as clinically indicated. The ovarian response to gonadotropins stimulation was monitored through ultrasound assessing both follicle sizes and follicle counts. Human Chorionic Gonadotropin (hCG) was administered ~36 h before the oocyte retrieval to induce ovulation. Oocyte collection was scheduled when follicle sizes reached 16–18 mm and embryo transfer (ET) was generally performed two to five days after the oocyte insemination according to embryo quantity and quality. In case of hypo-response or abnormal follicular growth, the cycle could be canceled before ovum pick up and these patients were not interviewed. However, they could be interviewed in a subsequent cycle reaching oocyte retrieval (to note, in women who were canceled for poor response, the subsequent cycle was not canceled again for this same reason and these women generally reached the oocyte retrieval). A freeze-all strategy was conversely preferred: (i) in case of hyper-response to reduce the risk of ovarian hyperstimulation syndrome (OHSS) (number of retrieved oocytes exceeding 15 or serum estradiol level exceeding 4000 pg/mL on the day of ovulation triggering), (ii) if serum progesterone exceeded 1500 pg/mL on the day of the ovulation triggering.

Couples underwent assisted reproduction with conventional in vitro fertilization or intracytoplasmatic sperm injection (ICSI) as clinically indicated and according to semen characteristics.

Qualified embryologists identified the total number of oocytes yielded per procedure, classified them as germinal vesicle or metaphase I or metaphase II (by the presence of a polar body) and determined fertilization 17–20 h after insemination (number of oocytes with two pronuclei). Good quality oocytes were those in metaphases I and II for IVF and metaphase II only for ICSI. Referring to embryological criteria, morphological features based on a stage-appropriate number of evenly sized blastomere, the absence/presence of multinucleation and the pattern of fragmentation were recorded after 48–72 h from activation (day 2 or 3). Embryos belonging to grade 1 or 2 (fragmentation less than 10% with equal-sized blastomeres) were classified as good quality ones [36].

Non-replaced viable embryos were vitrified mainly at the blastocyst stage. Patients with frozen embryos were scheduled for natural cycle embryo transfer if they reported regular menstrual cycles and a mean cycle length between 24 and 35 days. The procedure was performed 4–6 days after luteinizing hormone (LH) surge (detected with the use of urinary sticks) according to the embryo age. No luteal phase support was given. We prescribed a hormone replacement treatment if women had irregular menstrual cycles or if the monitoring of a natural cycle had previously failed.

After 14–16 days from the ovulation triggering or LH surge, women underwent blood hCG assessment to detect if implantation occurred and those with positive hCG values underwent a transvaginal ultrasound exam three weeks later. Clinical pregnancy was defined as the presence of at least one intrauterine gestational sac and live birth was defined as the birth of a viable newborn on or after 24 weeks of gestation.

All the clinical information (including infertility diagnoses, stimulation protocol type, gonadotropin dose and embryological data) was collected from medical and biological records.

### 2.3. Statistical Analysis

The primary objective was clinical pregnancy. Considering a 30% pregnancy rate per cycle, based on our Infertility Unit experience, this study was powered to detect a 1.5 increase of risk in the lowest quartile of glycemic load as compared to the highest (α = 0.05, β = 0.80).

We assessed multiple secondary outcomes in this analysis: (i) the number of retrieved good quality oocytes, (ii) the number of good quality embryos, (iii) the embryo transfer, (iv) the clinical pregnancy and (v) live birth.

Continuous variables were described as the mean and standard deviation (SD) if normally distributed, or median and interquartile range (IQR) if not normally distributed and they were analyzed using an analysis of variance and a Kruskal–Wallis test, respectively. Categorical variables were described as frequency (N) and percentage (%) and compared using the Pearson or Mantel–Haenzsel (MH) chi-square, as appropriate. We estimated the relative risk (RR) of each outcome and the corresponding 95% CI for the quartiles of the glycemic index, glycemic load, carbohydrate intake and intake of bread, pasta and fiber in the year before the interview. The RRs for whole grains and legumes consumption (regular users versus abstainers) were also calculated. In the multiple log-binomial regression model we included variables that were associated with at least one of the IVF outcomes in order to account for the potential confounders (as indicated in table footnotes). To control for type I error in a multiple testing situation, we applied the Multtest SAS Procedure with the Hochberg method [37,38].

All the analyses were performed using the SAS software, version 9.4 (SAS Institute, Inc., Cary, NC, USA).

## 3. Results

Out of 501 eligible women, seven (1.4%) were excluded from the analysis, as they did not provide complete information about their dietary habits. We observed no significant difference in the socio-demographic characteristics, clinical features and IVF outcomes between included and excluded subjects.

Table 1 shows the baseline and reproductive characteristics of the remaining 494 women included in the study. They had a mean age of 36.6 years (standard deviation, SD, 6.0, range 27–45) and a mean body mass index (BMI) of 22.4 kg/m^2^ (SD 4.0, range 17.0–42.0), with 29 women (5.9%) presenting a BMI in the obesity range (≥30 kg/m^2^). Most of the study subjects had a high education level (college degree, 51.6%), had never smoked (55.3%) and had previously undergone ART procedures (57.7%). The primary infertility diagnosis was the male factor (26.1%), followed by endometriosis (20.9%), low ovarian reserve (19.6%) and unexplained infertility (18.4%).

Overall, 429 of 494 (86.8%) underwent the embryo transfer, 158 of 494 (32.0%) achieved a clinical pregnancy and 121 of 494 (24.5%) had a live birth. Hence, 37 clinical pregnancies did not result in a live birth, of these, 34 ended in a miscarriage, one ended with an induced abortion, one was an ectopic pregnancy and one patient was lost during the follow-up (Figure S1).

Table 2 shows the clinical outcomes of IVF cycles according to dietary habits. In particular, the following dietary features were considered: glycemic load, daily carbohydrate intake, glycemic index, daily fiber intake and daily consumption of selected food groups rich in carbohydrates, like bread, pasta, whole grains and legumes. The study cohort was divided into quartiles of intake of each dietary component except for whole grains and legumes consumption.

No significant associations emerged between the considered exposures and the number of high-quality collected oocytes and the number of good-quality developed embryos.

We calculated the adjusted RRs for IVF failure at each step of the procedure (embryo transfer, clinical pregnancy, live birth) and no significant association was observed. The analysis included age, college degree, BMI, leisure physical activity and previous ART cycles in the model. Indeed, after adjusting for age, previous IVF cycles were associated to a higher risk of unavailability of embryos for the ET procedure with borderline significance (RR 1.58, 95% CI 0.96–2.59), while exercising for ≥5 h per week was associated to a higher risk of not achieving clinical pregnancy (RR 1.19, 95% CI 1.02–1.39) compared to exercising for <2 h per week.

Furthermore, since the consumption of vegetables and their dressings, in particular olive oil, may counteract the glycemic response of food, the association of the number of high quality oocytes and high quality embryos with the GL, carbohydrate intake, the GI and with the intake of bread and pasta was further analyzed in separate strata of vegetable intake, however no significant relation emerged (Table S1).

Finally, we investigated the role of GL, carbohydrate intake and GI in strata of cause of infertility (Table 3) and body mass index (BMI < 25 and BMI ≥ 25, Table S2). No significant relationship emerged in this further analysis, with the exception of a significant association between the GL and the number of good quality embryos in women with endometriosis (*p* = 0.02, for Kruskal–Wallis rank-sum): the number of good quality embryos decreased with an increasing quartile of glycemic load in women with endometriosis-related infertility. However, this finding lost significance after the Hochberg correction (*p* = 0.36 after correction).

## 4. Discussion

In this prospective Italian cohort of women undergoing in vitro fertilization we found no association between carbohydrate consumption, glycemic load, glycemic index, daily fiber intake and the daily intake of selected foods rich in carbohydrates and IVF outcomes, at each step of the procedure. Moreover, no significant relation emerged when the analysis was conducted in strata of vegetable intake, cause of infertility and BMI (BMI < 25 and BMI ≥ 25).

Although we are unaware of previous studies primarily investigating the association between carbohydrate intake, glycemic load and the outcomes of in vitro fertilization, several reports have to date showed that the amount and quality of carbohydrates in the diet may influence the fertility and ovulatory function, not only in PCOS and obese women (i.e., conditions characterized by impaired glucose metabolism and insulin resistance) but also in apparently healthy women [5,16,18]. However, the exact mechanism subtending the correlation between sugars and reproduction, in healthy premenopausal women, is still under investigation and available data present some inconsistency.

The Nurses’ Health Study II reported that high-carbohydrate diets with a high glycemic index were associated with the increased risk of infertility due to ovulatory disorders in apparently healthy women, while, as observed in our study, no relation emerged between the total fiber intake and the risk of anovulatory infertility [18].

Moreover, few studies have investigated the effectiveness of low glycemic index (LGI) diets and low carbohydrate diets (LCD) in improving reproductive outcomes in overweight/obese women and in PCOS women, compared with traditional, low-fat or high-carbohydrate diets. Marsh and colleagues conducted a study in 96 PCOS women and they observed that a modest weight loss (4%–5% of body weight) together with a moderate-carbohydrate diet with a low GI provided an improvement in the menstrual regularity and in reproductive outcomes compared with a conventional low-calorie diet [39]. Similarly, in a meta-analysis of eight randomized controlled trials (RCTs) including PCOS women, Zhang et al. concluded that, based on available evidences, low-carbohydrate diets (LCD) could effectively control body weight and they could restore, at least in part, insulin sensitivity, thus counteracting glucose metabolism impairment, gonadotropin imbalance and ovarian dysfunction in PCOS patients [20]. Becker and colleagues investigated the influence of a hypocaloric diet including low GI and low GL foods on metabolic variables and reproductive outcomes in a sample of overweight/obese infertile women undergoing IVF treatments [40]. In this small RCT, patients who were assigned to the low glycemic index diet group (cases) showed significantly better reproductive outcomes (i.e., improvement in the number of oocytes retrieved, pregnancy and live birth rates, both after natural and assisted conception) compared with the control group, who maintained its usual diet [40].

Conversely, other studies did not find any significant association between the dietary carbohydrate consumption, glycemic load and the plasma sex steroid levels [41,42].

The inconsistency observed in the results of the different studies may have various explanations. Considering the study by Chavarro et al. [18], this discrepancy may be partially due to some differences between the two study populations. Indeed, our study cohort was characterized by a higher mean age (37 years vs. 33 years), including both overweight/obese women and PCOS patients. Furthermore, some differences in dietary habits are worthy to be highlighted. Comparing GI values characterizing our study cohort with those of the US cohort described by Chavarro et al., the lower (56.0) and upper (74.2) ends of the range defining our first quartile of GI were much higher than the values characterizing the US population, where 56 and 58 represented the median GI of the fourth and the fifth quintile, respectively [18]. On the contrary, the daily total fiber intake was comparable between the two cohorts and in both studies no association was observed between the fiber consumption and the reproductive outcomes.

Moreover, in the study by Chavarro and colleagues [18], the primary endpoint was the risk of anovulatory infertility in a cohort of women who conceived or were trying to conceive spontaneously, while our analysis focused on women of infertile couples undergoing IVF. Thus, it is conceivable that assisted reproductive procedures could overcome some difficulties affecting natural conception, primarily, for example, oligo-anovulation, and therefore attenuating the influence of GL and GI on the reproductive prognosis.

Furthermore, a high vegetable intake may modify the glycemic response by various mechanisms (e.g., slowing carbohydrate absorption or reducing glucose toxicity to the pancreatic beta cells). Although in our study no difference emerged in the effect of GI, GL, carbohydrate intake and oocyte and embryo quality in strata of vegetable intake, it could be speculated that the effect of GL and GI on reproductive outcomes may be less evident in a cohort of Italian infertile women due to the mean higher intake of vegetables characterizing the Mediterranean–Italian dietary pattern compared to the US ones [43,44]. Indeed, our study population comes from a region where the Mediterranean diet is very common and it is conceivable that components of this dietary model, such as fiber, protein and organic acids, can modify the overall GL of the final meal, composed of various foods.

Some limitations of our study are worth considering. Although we used a previously validated questionnaire [25], self-reporting a diet is subject to measurement errors, it may not be sufficiently accurate and some misclassifications may have occurred. However, a dietary counseling is not routinely required in Italy before ART procedures, so the underreporting of unhealthy or unfavorable dietary habits should be unlikely. Furthermore, the FFQ was proved to be satisfactorily reproducible [23,24,45].

As this was an observational study, the possibility of residual sources of bias remains, including selection or confounding factors, which were not assessed or just poorly assessed in our study. However, if present, these biases are unlikely to have produced marked effects, especially considering that all the participants were interviewed by the same staff, in the same institution and that the participation rate was almost complete. Another potential limitation refers to the GI and GL values. Indeed, they may present some variability according to specific foods and cooking methods. For example, pasta and rice arouse different glycemic responses and they were assessed together in our FFQ. However, this information bias is unlikely to have a relevant impact on our estimates, as rice accounts for less than 20% of this food group in the study cohort [46]. The study power represents a further possible limitation, as with our sample size, comparison between the highest and the lowest quartile could identify a risk of pregnancy loss of about 1.5. Some issues of reliability in the assessment of physical activity may represent another limitation. Indeed, we used a score with no validation and no quantification of the total energy expenditure. Nonetheless, it has been shown that even simple questions on physical activity may provide reliable information [47]. Lastly, the study participants were infertile Italian women undergoing ART procedures, therefore our observations should not be equally generalized to the whole reproductive population nor to non-European couples.

## 5. Conclusions

In conclusion, we found no association between the dietary carbohydrate quantity and quality and the IVF outcomes in a cohort of infertile Italian women. The results of our study are not in line with previous evidences suggesting a negative effect of carbohydrate consumption and high glycemic load diet on fertility. Inconsistency among reports may be partially due to differences in dietary patterns and dietary habits among different populations. To our knowledge, this is the first study specifically focusing on the association of dietary carbohydrate quality and quantity with the outcomes of in vitro fertilization, including oocytes and embryo quality. Thus, in the light of the tight correlation between energy metabolism and female reproductive function, further researches involving the use of tools other than self-reporting food questionnaire and interventional analysis are required to investigate the role of dietary glycemic index and glycemic load in assisted reproduction. Moreover, considering that dietary patterns vary widely according to the geographical region, similar studies should be conducted in populations with different diet models. Robust evidences on this issue are of primary importance to design nutritional recommendations for infertile couples unabledergoing ART procedures.

## Figures and Tables

**Table 1:** Baseline and reproductive characteristics of the 494 included women.

	Number	%
Class Age (Years)		
Mean ± SD	36.6 ± 6.0	
<35	136	27.5
35–39	245	49.6
≥40	113	22.9
College Degree	255	51.6
BMI (kg/m^2^)		
<25	394	79.8
≥25	97	19.6
Smoking Habits		
Never	273	55.3
Current	91	18.4
Former	130	26.3
Caffeine Intake (Tertiles)		
1st	163	33.0
2nd	168	34.0
3rd	163	33.0
Alcohol Intake (Units/Week)		
Abstainers	140	28.3
<1	92	18.6
1–2	86	17.4
≥2	176	35.6
Occupational Physical Activity		
Heavy/moderate	139	28.1
Mainly standing	106	21.5
Mainly sitting	247	50.0
Leisure Physical Activity (hours/week)		
<2	266	53.8
2–4	176	35.6
≥5	51	10.3
Diagnosis of Infertility		
Male factor only	129	26.1
Endometriosis	103	20.9
Tubal	54	10.9
Low ovarian reserve	97	19.6
Ovulatory	20	4.0
Unexplained	91	18.4
Previous ART cycles	285	57.7
	**Median**	**IQR**
Total Calories Intake (kcal/die)	1707	1421–2036
FSH (mIU/mL)	7.3	5.8–8.9
AMH (ng/mL)	1.6	0.8–3.2

BMI: body mass index; IQR: interquartile range; ART: assisted reproduction techniques; FSH: follicle-stimulating hormone; AMH: anti-Muellerian hormone.

**Table 2:** Intermediate and clinical in vitro fertilization (IVF) outcomes according to the dietary habits of the 494 included women.

	N.	Number of Good-Quality Oocytes (Median, Q1–Q3)	Number of Good-Quality Embryos (Median, Q1–3)	Embryo Transfer		aRR (95% CI)	Clinical Pregnancy		aRR (95% CI)	Live Birth		aRR (95% CI)
				*No* = *65*	*Yes* = *429*		*No* = *336*	*Yes* = *158*		*No* = *372*	*Yes* = *121*	
**Glycemic Load**												
1st Quartile	123	5 (3–8)	1 (0–3)	15	108	1 *	83	40	1 *	92	30	1 *
2nd Quartile	123	5 (3–9)	2 (1–4)	16	107	1.29 (0.66–2.53)	79	44	0.99 (0.88–1.11)	89	34	0.99 (0.89–1.11)
3rd Quartile	124	5 (3–7)	2 (1–4)	17	107	1.41 (0.71–2.80)	82	42	0.99 (0.87–1.13)	92	32	1.00 (0.89–1.13)
4th Quartile	124	4 (2–7)	1 (0–3)	17	107	1.80 (0.80–4.05)	92	32	1.06 (0.92–1.21)	99	25	1.05 (0.92–1.20)
		*p* = *0.25*	*p* = *0.08*			*p* for trend *0.96*			*p* for trend *0.76*			*p* for trend *0.91*
**Carbohydrate Intake**												
1st Quartile	124	5 (3–8)	1 (0–3)	15	109	1 *	83	41	1 *	92	31	1 *
2nd Quartile	123	5 (3–9)	2 (1–4)	20	103	1.41 (0.76–2.63)	80	43	0.98 (0.88–1.11)	89	34	0.98 (0.87–1.10)
3rd Quartile	122	4 (3–7)	2 (1–4)	15	107	1.06 (0.55–2.07)	83	39	1.01 (0.90–1.14)	94	28	1.02 (0.91–1.14)
4th Quartile	125	4 (2–8)	2 (1–3)	15	110	1.08 (0.55–2.11)	90	35	1.04 (0.92–1.17)	97	28	1.02 (0.91–1.15)
		*p* = *0.57*	*p* = *0.10*			*p* for trend *0.30*			*p* for trend *0.89*			*p* for trend *0.97*
**Glycemic Index**												
1st Quartile (56.0–74.2)	124	5 (3–8)	2 (1–3)	16	108	1 *	85	39	1 *	92	31	1 *
2nd Quartile (74.3–77.1)	124	5 (3–7)	2 (1–4)	11	113	0.76 (0.37–1.57)	82	42	0.98 (0.86–1.13)	94	30	1.01 (0.91–1.13)
3rd Quartile (77.2–80.0)	123	4 (2–7)	1 (1–3)	18	105	1.26 (0.70–2.37)	83	40	1.00 (0.87–1.16)	93	30	1.01 (0.90–1.14)
4th Quartile (80.1–87.8)	123	5 (2–8)	2 (1–3)	20	103	1.35 (0.73–2.49)	86	37	1.01 (0.89–1.16)	93	30	1.00 (0.89–1.13)
		*p* = *0.20*	*p* = *0.88*			*p* for trend 0.24			*p* for trend 0.95			*p* for trend 0.96
**Bread**												
1st Quartile	119	5 (3–8)	2 (1–4)	13	106	1 *	79	40	1 *	87	32	1 *
2nd Quartile	112	4 (3–8)	1 (1–3)	17	95	1.48 (0.76–2.92)	67	45	0.96 (0.84–1.10)	77	34	0.98 (0.86–1.11)
3rd Quartile	138	5 (3–8)	2 (1–4)	16	122	1.19 (0.60–2.37)	98	40	1.05 (0.93–1.18)	109	29	1.06 (0.95–1.17)
4th Quartile	125	4 (2–7)	2 (0–3)	19	106	1.46 (0.76–2.83)	92	33	1.05 (0.94–1.19)	99	26	1.04 (0.93–1.16)
		*p* = *0.23*	*p* = *0.62*			*p* for trend 0.58			*p* for trend 0.38			*p* for trend 0.41
**Pasta**												
1st Quartile	123	5 (2–8)	1 (0–3)	19	104	1 *	92	31	1 *	96	27	1 *
2nd Quartile	112	5 (3–8)	2 (1–4)	18	94	1.12 (0.62–2.01)	75	37	0.94 (0.84–1.06)	87	24	1.00 (0.90–1.12)
3rd Quartile	138	5 (3–8)	2 (1–3)	12	126	0.66 (0.33–1.31)	90	48	0.95 (0.85–1.06)	97	41	0.96 (0.86–1.07)
4th Quartile	121	5 (3–7)	2 (1–3)	16	105	0.90 (0.49–1.66)	79	42	0.94 (0.84–1.05)	92	29	0.98 (0.88–1.09)
		*p* = *0.94*	*p* = *0.64*			*p* for trend 0.18			*p* for trend 0.11			*p* for trend 0.28
**Whole Grain**												
Abstainers	317	5 (3–8)	2 (1–3)	45	272	1 *	213	104	1 *	235	81	1 *
Regular users	177	5 (3–8)	2 (1–3)	20	157	0.77 (0.47–1.26)	123	54	1.01 (0.91–1.12)	137	40	1.02 (0.93–1.11)
		*p* = *0.81*	*p* = *0.38*									
**Legumes**												
Abstainers	169	5 (3–8)	1 (1–3)	22	147	1 *	118	51	1 *	126	42	1 *
Regular users	325	5 (3–8)	2 (1–3)	43	282	0.99 (0.62–1.60)	218	107	0.96 (0.88–1.06)	246	79	0.99 (0.91–1.07)
		*p* = *0.16*	*p* = *0.24*									
**Fiber Intake (g/d)**												
1st Quartile (5.50–13.30)	122	5 (3–8)	1 (0–4)	19	103	1 *	79	43	1 *	88	34	1 *
2nd Quartile (13.31–16.86)	124	5 (3–7)	2 (1–3)	15	109	0.73 (0.39–1.36)	86	38	1.02 (0.89–1.18)	98	25	1.05 (0.93–1.19)
3rd Quartile (16.87–0.85)	123	5 (2–8)	2 (1–3)	17	106	0.84 (0.46–1.53)	85	38	1.02 (0.89–1.18)	93	30	1.01 (0.89–1.15)
4th Quartile (20.86–40.10)	125	5 (3–7)	2 (1–4)	14	111	0.69 (0.36–1.31)	86	39	1.03 (0.89–1.19)	93	32	1.01 (0.89–1.15)
		*p* = *0.90*	*p* = *0.54*			*p* for trend 0.10			*p* for trend 0.82			*p* for trend 0.52

aRR: adjusted relative risk; CI: confidence interval; BMI: body mass index, PA: physical activity. * Reference category. The final model includes age class (<35, 35–39, ≥40), college degree, BMI class (<25.0, ≥25.0), previous ART cycles (no, yes) and leisure PA.

**Table 3:** Relation between the glycemic load (GL), carbohydrate intake, the glycemic index (GI) and the number of high-quality oocytes and embryos in strata of cause of infertility.

	Male Factor Only		Endometriosis		Tubal		Low Ovarian Reserve		Ovulatory		Unexplained	
	Median (IQR)		Median (IQR)		Median (IQR)		Median (IQR)		Median (IQR)		Median (IQR)	
	Number of Good-Quality Oocytes	Number of Good-Quality Embryos	Number of Good-Quality Oocytes	Number of Good-Quality Embryos	Number of Good-Quality Oocytes	Number of Good-Quality Embryos	Number of Good-Quality Oocytes	Number of Good-Quality Embryos	Number of Good-Quality Oocytes	Number of Good-Quality Embryos	Number of Good-Quality Oocytes	Number of Good-Quality Embryos
**Glycemic Load**												
1st Quartile	6 (4–10)	1 (0–4)	3 (1–8)	1 (0–3)	4 (2–8)	1 (1–4)	3 (2–5)	1 (0–2)	8 (4–11)	2 (2–4)	7 (6–8)	3 (2–3)
2ndQuartile	7 (3–9)	1 (1–4)	5 (3–8)	1 (1–4)	8 (3–9)	3 (1–7)	3 (2–4)	1 (1–2)	5 (3–11)	2 (1–5)	7 (5–10)	2 (1–6)
3rd Quartile	6 (3–10)	2 (1–4)	4 (3–6)	2 (0–3)	7 (4–7)	3 (2–4)	3 (2–5)	2 (1–2)	7 (5–12)	2 (0–3)	7 (3–8)	2 (1–3)
4th Quartile	5 (2–9)	1 (0–3)	3 (2–6)	0 (0–2)	4 (2–11)	2 (1–4)	4 (2–6)	2 (1–3)	5 (4–6)	3 (2–3)	6 (2–9)	2 (1–4)
*P*	0.76	0.24	0.11	0.02	0.62	0.42	0.25	0.16	0.50	0.95	0.49	0.71
**Carbohydrate Intake**												
1st Quartile	6 (4–10)	1 (0–3)	3 (2–8)	1 (0–2)	6 (3–9)	1 (1–4)	2 (2–5)	1 (0–2)	8 (4–11)	2 (2–4)	7 (6–8)	3 (1–4)
2nd Quartile	5 (3–9)	1 (1–4)	5 (3–9)	2 (1–4)	6 (3–8)	3 (2–7)	3 (2–5)	1 (0–2)	5 (3–11)	2 (1–5)	8 (6–11)	2 (1–6)
3rd Quartile	7 (3–10)	2 (1–4)	4 (3–6)	2 (0–3)	7 (4–7)	3 (2–4)	3 (2–4)	2 (1–2)	7 (5–12)	2 (0–3)	6 (3–8)	2 (1–3)
4th Quartile	6 (3–9)	1 (0–4)	3 (2–6)	1 (0–3)	4 (2–10)	3 (1–3)	3 (2–6)	2 (1–3)	5 (4–6)	3 (2–3)	6 (2–9)	2 (1–3)
*P*	0.94	0.42	0.49	0.13	0.94	0.29	0.24	0.24	0.50	0.95	0.23	0.80
**Glycemic Index**												
1st Quartile	7 (5–10)	1 (1–5)	4 (3–7)	1 (1–3)	5 (3–8)	3 (1–4)	2 (2–4)	1 (0–2)	8 (4–11)	2 (2–4)	8 (6–10)	3 (2–5)
2ndQuartile	6 (3–10)	1 (1–4)	4 (3–7)	1 (0–3)	8 (5–9)	4 (3–5)	3 (3–5)	1 (0–3)	5 (3–6)	3 (2–3)	7 (5–7)	2 (0–3)
3rd Quartile	5 (3–9)	1 (1–3)	3 (2–9)	1 (0–2)	4 (2–8)	2 (1–5)	3 (2–5)	2 (1–2)	6 (4–11)	2 (1–3)	5 (3–8)	2 (1–3)
4th Quartile	5 (2–9)	1 (0–3)	4 (3–6)	2 (0–3)	6 (4–10)	3 (1–3)	5 (2–6)	2 (0–3)	5 (4–6)	3 (3–3)	8 (4–11)	2 (1–5)
*P*	0.38	0.86	0.98	0.94	0.43	0.53	0.17	0.40	0.72	0.44	0.14	0.18

IQR: Inter Quartile Range.

## Supplementary Materials

The following are available online at https://www.mdpi.com/2072-6643/12/6/1568/s1. Figure S1: Flow Chart, Table S1: Relation between GL, carbohydrate intake, GI and the number of high-quality oocytes and embryos in strata of vegetable intake. Table S2: Relation between GL, carbohydrate intake, GI and the number of high-quality oocytes and embryos in strata of BMI.
