# Supplementary material for: Dietary Carbohydrate Intake, Dietary Glycemic Load and Outcomes of In Vitro Fertilization: Findings from an Observational Italian Cohort Study
Source: Nutrients. 2020 May 28;12(6):1568. doi: 10.3390/nu12061568 (PMC7352402; doi:10.3390/nu12061568)

nutrients MDPI

Table S1. Relation between GL, Carbohydrate Intake, GI and the number of high-quality oocytes and embryos in strata of vegetable intake.

	Vegetable Intake < 13 servings/week						Vegetable intake ≥ 13 servings/week					
	N. of good-quality oocytes			N. of good-quality embryos			N. of good-quality oocytes			N. of good-quality embryos		
	Median	Q1	Q3	Median	Q1	Q3	Median	Q1	Q3	Median	Q1	Q3
**Glycemic Load**												
1st Quartile	5.0	2.0	7.0	1.0	0.0	3.0	5.0	3.0	8.0	1.0	0.0	2.0
2nd Quartile	5.0	3.0	8.0	2.0	1.0	4.0	6.0	3.0	9.0	1.0	1.0	3.0
3rd Quartile	4.0	3.0	7.0	2.0	0.0	3.0	5.0	3.0	8.0	2.0	1.0	4.0
4th Quartile	4.0	2.0	6.0	1.0	0.0	3.0	5.0	2.0	7.0	1.0	0.0	3.0
**Carbohydrate Intake (g/d)**												
1st Quartile	5.0	2.0	7.0	1.0	0.0	3.0	5.0	3.0	9.0	1.0	0.0	2.0
2nd Quartile	5.0	3.0	7.0	1.0	0.0	3.0	6.0	3.0	10.0	2.0	1.0	4.0
3rd Quartile	4.0	3.0	7.0	2.0	1.0	4.0	5.0	3.0	7.0	2.0	1.0	4.0
4th Quartile	4.0	2.0	7.0	1.5	0.5	3.0	5.0	2.0	8.0	1.0	0.0	3.0
**Glycemic Index**												
1st Quartile	6.0	4.0	8.0	2.0	1.0	4.0	5.0	3.0	8.0	1.0	0.0	3.0
2nd Quartile	4.0	3.0	7.0	1.0	0.0	3.0	6.0	3.0	7.0	2.0	1.0	4.0
3rd Quartile	4.0	2.0	6.5	1.5	1.0	3.0	5.0	2.0	9.0	1.0	0.0	2.0
4th Quartile	4.5	2.0	7.5	2.0	0.0	3.0	5.0	2.0	9.0	2.0	1.0	4.0

Q: Quartile.

Table S2. Relation between GL, Carbohydrate Intake, GI and the number of high-quality oocytes and embryos in strata of BMI.

	BMI < 25 (Kg/m^2)						BMI ≥ 25 (Kg/m^2)					
	N. of good-quality oocytes			N. of good-quality embryos			N. of good-quality oocytes			N. of good-quality embryos		
	Median	Q1	Q3	Median	Q1	Q3	Median	Q1	Q3	Median	Q1	Q3
**Glycemic Load**												
1st Quartile	5.0	3.0	8.0	1.0	0.0	3.0	4.0	2.0	7.0	1.0	0.0	3.0
2nd Quartile	5.0	3.0	9.0	2.0	1.0	4.0	5.0	3.0	10.0	1.0	1.0	3.0
3rd Quartile	5.0	3.0	7.0	2.0	0.0	3.0	4.0	3.0	7.0	2.0	2.0	4.0
4th Quartile	4.0	2.0	7.0	1.0	0.0	3.0	5.0	2.0	9.0	1.0	0.0	3.0
**Carbohydrate Intake (g/d)**												
1st Quartile	5.0	3.0	8.0	1.0	0.0	3.0	4.0	2.0	7.0	1.0	0.0	3.0
2nd Quartile	5.0	3.0	9.0	2.0	1.0	4.0	4.5	3.0	10.5	1.5	0.5	4.5
3rd Quartile	4.0	3.0	7.0	2.0	0.0	3.0	4.0	3.0	7.0	2.0	1.0	4.0
4th Quartile	4.0	2.0	7.0	1.5	0.0	3.0	5.0	2.0	9.0	1.0	1.0	3.0
**Glycemic Index**												
1st Quartile	6.0	3.0	8.0	2.0	1.0	3.0	3.5	2.0	7.5	1.0	0.0	2.5
2nd Quartile	4.0	3.0	7.0	1.0	0.0	3.0	6.0	4.0	9.0	2.0	0.0	4.0
3rd Quartile	4.0	2.0	7.0	1.0	1.0	3.0	3.5	2.0	7.5	1.0	0.0	3.0
4th Quartile	5.0	2.0	8.0	2.0	0.0	3.0	4.5	3.0	7.5	2.0	1.0	3.0

Q: Quartile.

Figure S1. Flow Chart. FFQ: Food Frequency Questionnaire; ET: Embryo Transfer

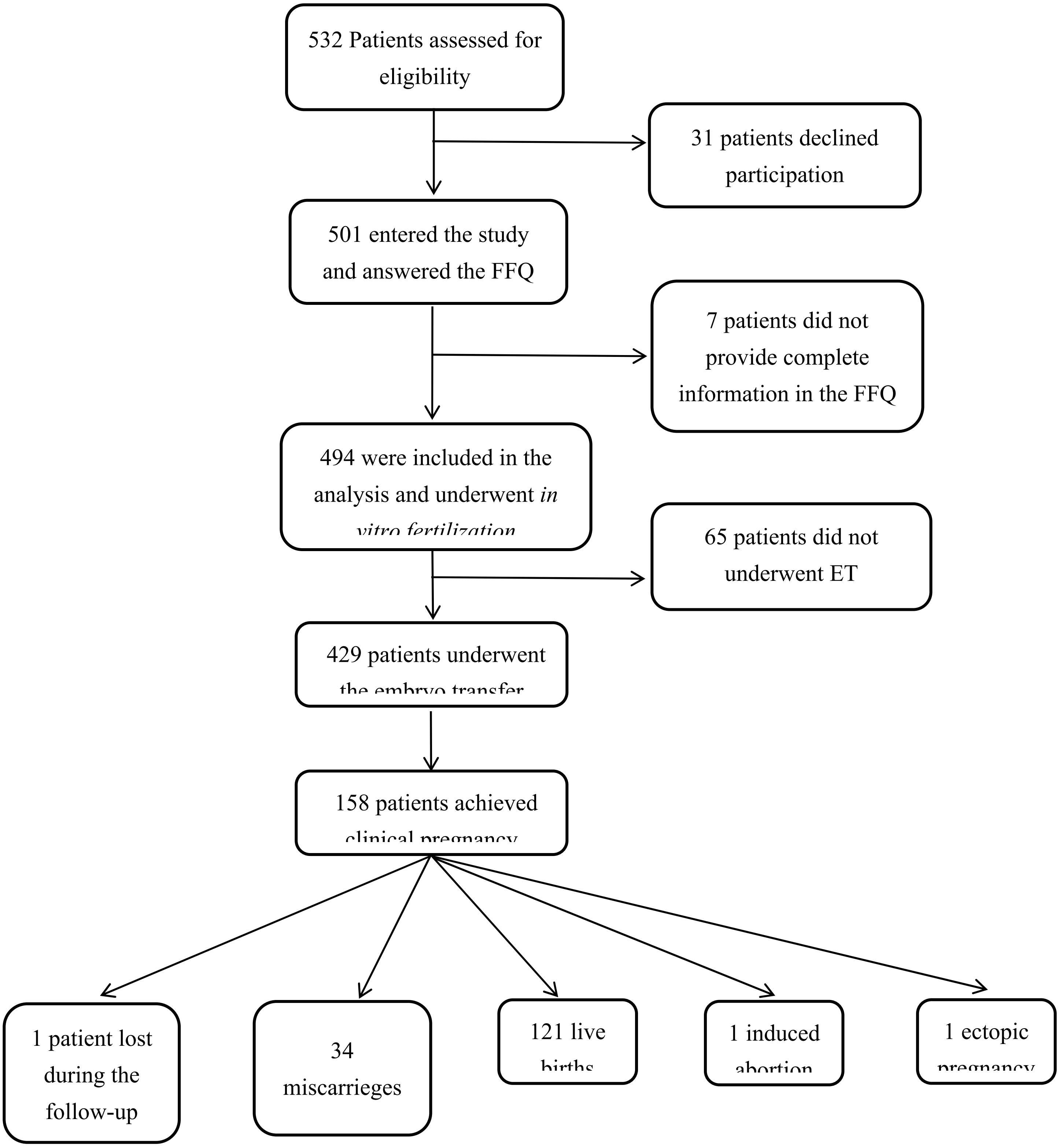

Supplement: Supplementary file 1 [file nutrients-12-01568-s001.pdf]
